# Comprehensive implementations of multiple imputation using retrieved dropouts for continuous endpoints

**DOI:** 10.1186/s12874-025-02494-5

**Published:** 2025-02-21

**Authors:** Shuai Wang, Pamela F. Schwartz, James P. Mancuso

**Affiliations:** Pfizer Research & Development, Pfizer Inc, New York, NY USA

**Keywords:** Multiple imputation, Retrieved dropouts, Treatment policy estimand, Phase 3 clinical trials, Chronic weight management, Type 2 diabetes

## Abstract

**Background:**

In the metabolic disease area, there has been a long-time debate against using mixed models for repeated measures (MMRM) as the primary analysis of longitudinal continuous endpoints. As missing data arising from missing not at random assumptions are not addressed in MMRM, multiple imputation based on specific assumptions has been brought into play. Among many missing not at random assumptions with varying degrees of conservativeness, multiple imputation based on retrieved dropouts (MIRD) has been accepted by regulatory agencies in several type 2 diabetes and chronic weight management products in recent years, marking the beginning of a new standard for analysis of longitudinal data in this disease area.

**Methods:**

On top of the established MIRD approach of which the imputation is based on last on-treatment data of retrieved dropout (RD)s, we propose a new class of MIRD approaches utilizing all available data from RDs. The imputation implementation can be one-step Markov Chain Monte Carlo (MCMC) or two-step (creating monotone missingness, followed by regression approach). ANCOVA can be applied to the complete dataset post imputation and Rubin’s rule can be used to combine all estimates into a single estimate. Simulation studies in a wide range of scenarios are conducted to understand the type-I error and power rates of the new class versus the established MIRD approach and other reference statistical methods such as MMRM.

**Results:**

Overall, the new class has very similar performance compared to the established MIRD approach based on last on-treatment data. What’s more interesting is the one-step MCMC approach has better controlled type-I error and is more powerful than the established MIRD approach in certain scenarios with the difference gradually diminishing with larger sample size. The data analyses based on two real phase 3 datasets further manifest the power conclusions, with the results based on the new class applied to the larger of the two datasets almost identical to that of on-study MMRM.

**Conclusions:**

We have presented comprehensive implementations of the MIRD approach for continuous endpoints in a longitudinal setting that fully fit within the strategy of treatment policy. The proposed new class based on all observed data of RDs is proved to be as powerful as the established MIRD approach based on last on-treatment visit in most scenarios. The one-step MCMC approach is more powerful than the established MIRD approach in certain scenarios. Since the new class involves less programming derivation of additional flags, it’s anticipated to be more easily implemented in clinical trial reporting.

**Supplementary Information:**

The online version contains supplementary material available at 10.1186/s12874-025-02494-5.

## Background

Retrieved dropout (RD) analyses have become the established primary analysis in the field of type 2 diabetes (T2D) and obesity/chronic weight management (CWM) in recent years. Under the guidance of regulatory agencies, recent phase 3 clinical programs of GLP-1 receptor agonists [1–17] have all implemented this approach with estimated treatment effect presented in the labels [18–20].

The mixed model for repeated measurements (MMRM) has long been regarded as the gold standard for analyzing longitudinal clinical trial data with proven superiority to several peers [21, 22]. However, there has been much debate against using MMRM [23, 24]. It has been noted that it’s incapable of handling situations of missing not at random (MNAR). Furthermore, the MMRM approach aligns with the hypothetical estimand [25, 26] which addresses the efficacy of the drug should the study participants not experience any intercurrent events. As it typically excludes all data that occur after intercurrent events from the analysis and uses an underlying missing at random (MAR) assumption to implicitly impute the missing data, it may not reflect the treatment effect in the real-world setting. Furthermore, although MMRM is sometimes applied to all available data, including data collected after intercurrent events, this results in the model using a mix of 2 different types of participants, adherent completers and RDs, to implicitly infer the likely outcomes for study dropouts with missing data, even though dropouts are logically expected to behave more like RDs than adherent completers.

The treatment policy estimand, on the other hand, accounts for the impact from intercurrent events by including all records that occur after intercurrent events in the analysis. It represents an average treatment effect across different participant adherence scenarios ranging from good to bad. There are a variety of statistical methods that fit in this paradigm with each of them usually driven by a unique MNAR assumption. Some assumptions tend to put more penalty on the dropouts, e.g. assuming the treatment effect will be completely washed out and their values at the “virtual” primary visit will fall back to baseline level or assuming the post-discontinuation values of the dropouts in the active treatment group will follow the distribution of the reference group [27, 28]; other assumptions are milder: for instance, assuming no further improvement once participants discontinue, which is similar to last observation carried forward (LOCF). In terms of implementation, there has been an increasing popularity to use multiple imputation (MI) over single imputation to characterize any specific assumption. MI has proved to be a powerful tool when the amount of missing data is non-negligible by means of between-imputation and within-imputation variances [29].

Because MI analyses are essentially driven by the underlying assumption regarding missing data, the sponsor must reach consensus with the regulatory agency on the intended assumption for pivotal trials. In this paper, we propose a class of methods that fall under the same MNAR assumption, in which participants with missing data are assumed to follow the same distribution as RDs (generally defined as participants who remain in the study despite treatment discontinuation). The reason that this type of analysis has become more and more appealing is its assumption is relatively neutral and both sponsors and regulatory agencies find it acceptable. There has been an established approach to implement this MIRD analysis [30]. However, because this implementation requires extensive programming derivations in real applications, the implementation may be confronted with challenges. We propose alternative implementations which will greatly simplify the process. To evaluate and compare the performance, we use simulation studies to assess the type-I error and power rates across different clinical scenarios. Results show all newly proposed approaches are very comparable to the established MIRD approach but with ease of implementation. It’s noteworthy among all MIRD approaches including the established MIRD approach, the one-step MCMC approach has the most robust type-I error control and is the most powerful. For certain study design scenarios, it’s more powerful than the established MIRD approach. In the data analysis section, we present real applications to two phase 3 studies with percent change from baseline in LDL at Week 52 as the primary endpoint of interest. Results are interpreted and compared to those of MMRM and return to baseline (RTB). Finally in the discussion section, all findings are summarized and concluded, and pros and cons of this approach are further discussed. Thoughts on potentially expanding the scope of RDs are also explored. Because binary endpoints in clinical studies are usually directly converted from continuous endpoints, the proposed approaches can be readily generalized to binary endpoints. Given that the framework for time to event endpoints is already in place [31], this topic with the underlying RD assumption is considered complete for all major types of endpoints for clinical studies.

## Methods

The following four MI methods based on RDs are explored and compared (Table 1). For all methods, missing data will be imputed by treatment group to ensure good imputation precision with no loss of generality. For instance, if the study has two arms with $p$ and $d$ denoting placebo and investigational drug respectively, there will be two separate multiple imputations, i.e., within ${{\varvec{\Omega}}}_{T} (T=p, d)$, a union of participants with missing data and RDs written as ${{\varvec{\Omega}}}_{T}={\mathbf{M}}_{T}\cup {\mathbf{R}}_{T}$ with ${\mathbf{M}}_{T}$ denoting participants with missing data and ${\mathbf{R}}_{T}$ denoting RDs, generally defined as participants who prematurely discontinue the treatment but remain in the study for follow-up through the primary visit. **R**_*T*_ is constructed as a union of RD subsets of participants who are in the same treatment group but discontinue treatment at different time points (i.e., ${\mathbf{R}}_{T }={\mathbf{R}}_{T1}\cup {\mathbf{R}}_{T2}\cup \dots \cup {\mathbf{R}}_{Tk}$, assuming there is a total of $k$ post-baseline visits prior to the primary visit). The missing data ${\mathbf{M}}_{T}$ is simply defined as participants in the treatment group $T$ who have dropped out of the study prior to the primary visit or have missing values at the primary visit due to other reasons. For analysis purposes, completers are defined as ${\varvec{C}}={\bigcup }_{T=p,d}{{\varvec{C}}}_{T}$, i.e., the rest of the participants after excluding ${\varvec{\Omega}}={\bigcup }_{T=p,d}{{\varvec{\Omega}}}_{T}$ from the original dataset [30]. Specifically, the completers are on-treatment completers whereas off-treatment completers are RDs according to the definition.

**Table 1:** Summary of the 4 MIRD methods

Name of the method	Short name (in the outputs)	# MI steps	Analysis Method post imputation
***Newly proposed*** assumes the primary visit depends on baseline and all observed intermediate visits in the imputation steps			
Class 1 (a): Two-step approach	Full_ANCOVA	2	ANCOVA
Class 1 (a): Two-step approach	Full_MMRM	2	MMRM
Class 1 (b): One-step MCMC approach	Full_MCMC	1	ANCOVA
***Existing*** assumes the primary visit depends on baseline and last on-treatment visit in the imputation steps			
Class 2: Established MIRD approach	Last_ANCOVA	1	ANCOVA

Every approach follows a process of imputation, analysis (“estimation”), and combination (“hypothesis testing”) [30]. The combination step is common to all approaches, but the imputation and analysis steps can be unique and are described as follows and summarized in Table 1. In this paper, participants with missing baseline values will be excluded from the analysis set.

**Class 1**. Use all observations from RDs including both on-treatment and post-treatment records as basis to impute the missing data. We start with introducing the two-step approach (a), followed by the one-step approach (b).

**Fig. 1:**
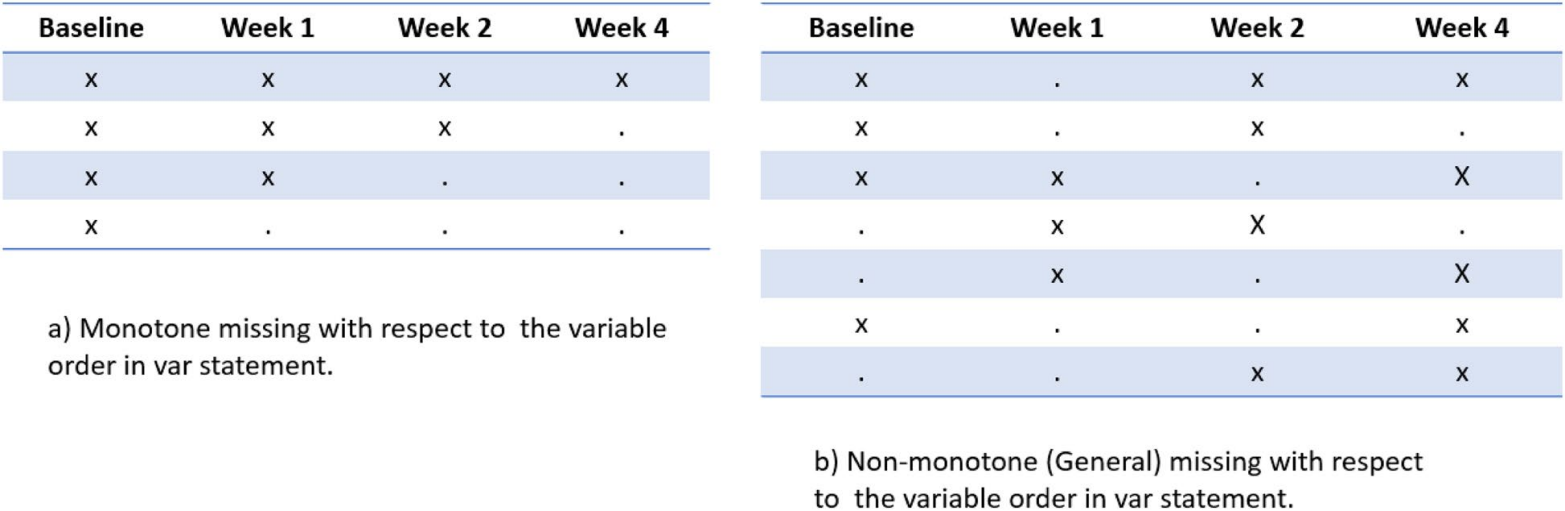
Monotone missing data pattern vs general missing pattern in trials with longitudinal visits

**Class 1 (a) (i.e., Two-step approach)**: Apply a two-step MI approach to impute the missing data of ${{\varvec{\Omega}}}_{T}$:

1. Firstly, out of ${{\varvec{\Omega}}}_{T}$, create $M$ (e.g., 100) datasets with monotone missing patterns (Fig. 1. a)) with respect to the visits in the longitudinal order, i.e., ${{\varvec{Y}}}_{b}$, ${{\varvec{Y}}}_{inter}$, ${\varvec{Y}}$**,** where ${{\varvec{Y}}}_{b}$ denotes baseline, ${{\varvec{Y}}}_{inter}$ and ${\varvec{Y}}$ denote intermediate post-baseline visits and primary visit, respectively. The monotone missingness is usually produced with MCMC [32, 33].
2. i. Apply linear regression models to the $M$ datasets to impute missing values of ${{\varvec{Y}}}_{inter}$ (where ${{\varvec{Y}}}_{inter}=[{{\varvec{Y}}}_{inter,1}, {{\varvec{Y}}}_{inter,2}, \dots , {{\varvec{Y}}}_{inter,q}]$) sequentially according to linear regression models (1), so that all missing values of predictors in model (2) are imputed.
  ii. Finally apply the regression model (2) to impute the missing data of ${\varvec{Y}}$, adjusting for baseline ${{\varvec{Y}}}_{b}$ and all subsequent visits ${{\varvec{Y}}}_{inter}$ prior to the primary visit.
  $${{\varvec{Y}}}_{inter,1}={\beta }_{0, T,1}^{IMP}+{\beta }_{1, T,1}^{IMP}{{\varvec{Y}}}_{b}+{\varvec{\varepsilon}}$$
  $${{\varvec{Y}}}_{inter,2}={\beta }_{0, T,2}^{IMP}+{\beta }_{1, T,2}^{IMP}{{\varvec{Y}}}_{b}+{\beta }_{2, T,2}^{IMP}{{\varvec{Y}}}_{inter,1}+{\varvec{\varepsilon}}$$
  $$\vdots$$
  1 $${{\varvec{Y}}}_{inter,q}={\beta }_{0, T,q}^{IMP}+{\beta }_{1, T,q}^{IMP}{{\varvec{Y}}}_{b}+\dots +{\beta }_{q, T,q}^{IMP}{{\varvec{Y}}}_{inter,(q-1)}+{\varvec{\varepsilon}}$$
  2 $${\varvec{Y}}={\beta }_{0, T}^{IMP}+{\beta }_{1, T}^{IMP}{{\varvec{Y}}}_{b}+{{\varvec{Y}}}_{inter}{{\varvec{\beta}}}_{2, T}^{IMP}+{\varvec{\varepsilon}}$$

Although theoretically step 2 is implemented in two sub steps sequentially, they are usually programmed within one Proc MI step using SAS. The missing data in the two sequential sub-steps are imputed by firstly sampling the regression coefficients from posterior predictive distribution of ${{\varvec{\beta}}}_{*, *}^{IMP}$ in the form of a multivariate normal distribution [34] using parameter estimates ${\widehat{{\varvec{\beta}}}}_{*, *}^{IMP}$ and associated covariance estimates, and then fitting the above equations.

Then one of the following analysis models will be used to analyze each of the $M$ imputed datasets after combined with ${\varvec{C}}$. Because each method is applied to an imputed dataset with no missing values, the results of the following two are expected to be equivalent for the primary visit, except for very small samples [35, 36].

- ◦ ANCOVA
- ◦ MMRM

**Class 1 (b) (i.e., One-step MCMC approach)**: Apply a one-step MI approach to ${{\varvec{\Omega}}}_{T}$ to impute missing data via MCMC Gibbs sampling, which doesn’t require monotone missingness (Fig. 1b). It handles different missing data patterns via predictive distribution $P({{\varvec{Y}}}_{miss}^{\left(u\right)}|{{\varvec{Y}}}_{obs},{\varvec{\theta}})$ assuming there is a total of $U$ missing data patterns ($u=1, \dots , U)$. The MCMC approach will alternate between missing data sampling (step 3) and parameter sampling from posterior distribution (step 2) until convergence is achieved [37, 38]. Specifically, the imputation is conducted as follows:

- *step 1*: Assign initial values to ${{\varvec{Y}}}_{miss};$
- *step 2* (P-step): Simulate ${\varvec{\theta}}$ from posterior distribution $P\left({\varvec{\theta}}|{{\varvec{Y}}}_{obs},{{\varvec{Y}}}_{miss}\right);$
- *step 3* (I-step): Draw ${{\varvec{Y}}}_{miss}^{\left(u\right)}$ from *conditional predictive distribution* $P\left({{\varvec{Y}}}_{miss}^{\left(u\right)}|{{\varvec{Y}}}_{obs},{\varvec{\theta}}\right)$ $(u=1, \dots , U);$
- *step 4*: Repeat step 2 and 3 until convergence.

Because imputations drawn from successive iterations on the same chain tend to be correlated, multiple chain or a single chain (keep every n-th iteration and discard the rest) should be considered to generate multiple imputations. ANCOVA is then used to analyze each of the $M$ complete datasets. 

**Class 2** (the established MIRD approach): Use the last on-treatment observations from RDs to impute the missing data. Specifically, a regression model based on ${\mathbf{R}}_{T}$ is constructed as follows, with ${\varvec{Y}}$**,** ${{\varvec{Y}}}_{b}$ and ${{\varvec{Y}}}_{L}$ denoting primary visit, baseline visit and last on-treatment visit respectively.

3 $${\varvec{Y}}={\beta }_{0, T}^{IMP}+{\beta }_{1, T}^{IMP}{{\varvec{Y}}}_{b}+{\beta }_{2, T}^{IMP}{{\varvec{Y}}}_{L}+{\varvec{\varepsilon}}$$

The imputation steps will be implemented by sampling regression coefficients and random error term from posterior predictive distribution and then fitting the regression equation for participants in ${\mathbf{M}}_{T}$ [30] for a total of $M$ times. Then ANCOVA is used to analyze each of the $M$ complete datasets.

For all four methods described above, Rubin’s rule [34] will be used to combine all estimates of treatment effect (e.g. ${\widehat{\beta }}_{2}^{(m)}$ from ANCOVA) into a single estimate which then will be tested by means of a total variance defined as the sum of within and between imputation variances, and a t-test statistic.

4 $$\begin{array}{cc}{{\varvec{Y}}}^{(m)}={\beta }_{0}^{(m)}+{{\beta }_{1}^{(m)}{\varvec{Y}}}_{b}+{\beta }_{2}^{(m)}{\varvec{t}}{\varvec{r}}{\varvec{t}}&(m=1, \cdots, M)\end{array}$$

## Simulations

In this section, we describe how simulation studies are conducted to compare the MIRD methods as well as other commonly used statistical methods. Starting from type-I error simulations, a variety of different clinical scenarios are generated under the null hypothesis to assess if the type-I error rate is controlled. Then we move to power simulations to assess the power rate with respect to different clinical scenarios (sample size, dropout rate and RD rate) assuming the drug is more effective than the comparator.

### Type-I Error

Datasets for Type-I simulations are generated at the null hypothesis that there’s no difference of treatment effect at the primary visit (i.e., ${{\varvec{\beta}}}_{eff}=0$). Different study dropout rates (i.e., missing rates) were simulated: 10%, 20%, 30%. Assuming these trials are designed to collect retrieved dropouts, we firstly evaluate the type-I error when retrieved dropout rate is close to the study dropout rate. Specifically, given a study dropout rate (i.e. missing rate), the following retrieved dropout rates are studied:

- RD rate = Missing rate – 5%
- RD rate = Missing rate
- RD rate = Missing rate + 5%

In each simulation, a 26-week two-armed (1:1) T2D trial with a total number of $N$ participants are created with baseline, week 6, 12, 18, 26 (primary visit) as planned site visits. All visits of HbA1c values (in %, the lower the better) including baseline are simulated from a multivariate normal distribution using a cLDA model [39]:

$$\begin{array}{cc}{{\varvec{Y}}}_{i}={{\varvec{\beta}}}_{0}+{{\varvec{\beta}}}_{t}+{{\varvec{\beta}}}_{eff}*{trt}_{i}+{\varvec{\varepsilon}}&(i=1,\cdots, N)\end{array}$$

where ${{\varvec{\beta}}}_{0}$ is the vector of baseline values, ${{\varvec{\beta}}}_{t}$ reflects the change from baseline over time, under placebo effect. ${trt}_{i}$ is randomized treatment group with 1 denoting active and 0 denoting placebo. ${{\varvec{\beta}}}_{eff}$ is the difference in treatment effect between active and placebo over time and ${\varvec{\varepsilon}}$ is the random vector representing correlation of visits among the same participant which follows a multivariate normal distribution specified in the supplementary file.

$${{\varvec{\beta}}}_{0}=(8.25, 8.25, 8.25, 8.25, 8.25)^{\prime}$$

$${{\varvec{\beta}}}_{t}=(0, -0.01, -0.05, -0.1, -0.2)^{\prime}$$

$${{\varvec{\beta}}}_{eff}=0$$

**Fig. 2:**
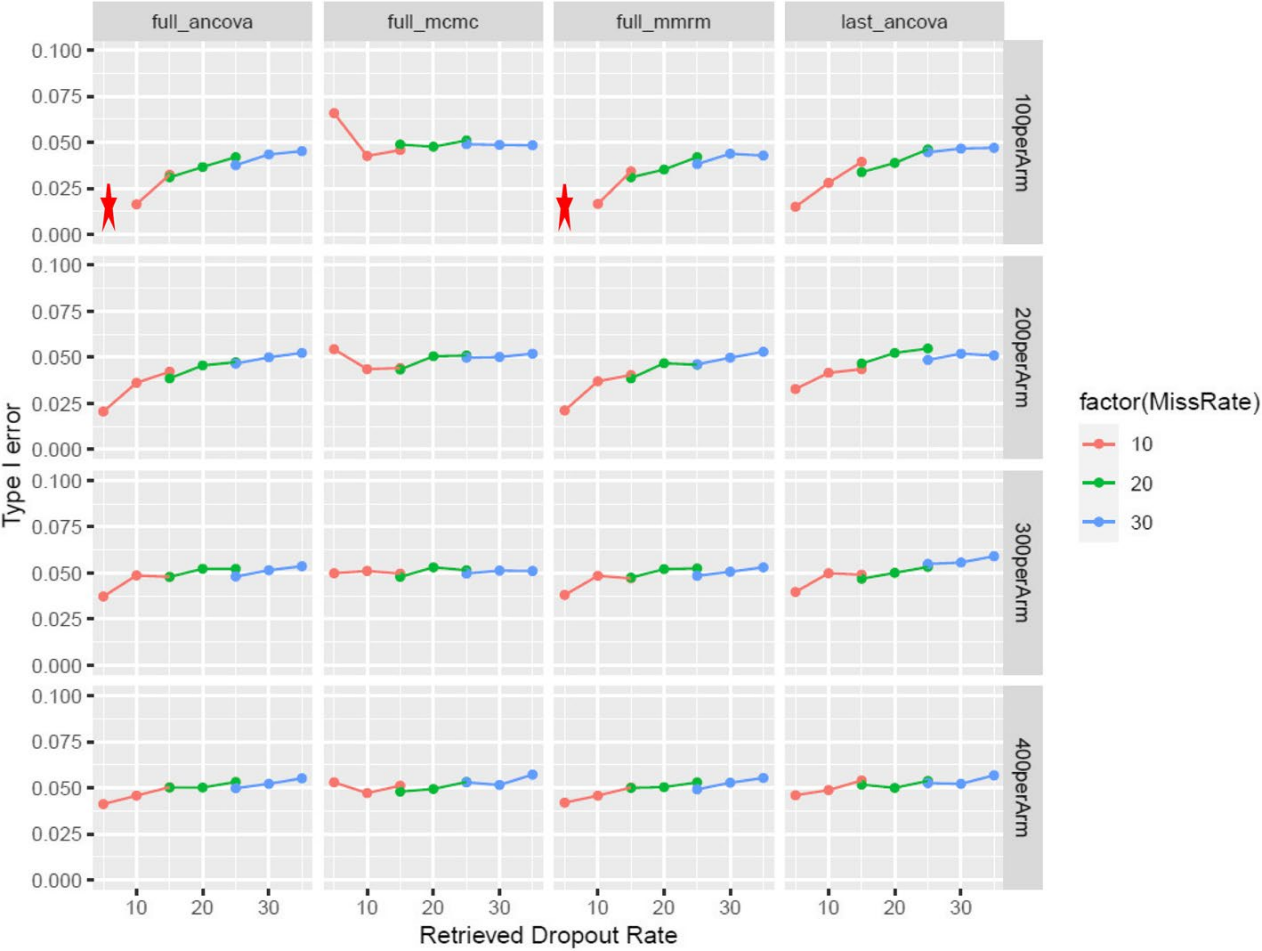
The type-I error rate for a wide variety of scenarios: the star denotes “not estimable” in which at least one simulation is not estimable, and therefore type-I error is not provided

Different sample size scenarios are simulated to understand the impact of sample size on type-I error rate and applicability (Table 2). For each scenario, 5000 datasets are simulated. Type-I error rate is summarized as proportion of simulations that are significant at a two-sided alpha of 0.05 at Week 26. The type-I error rate and bias for all scenarios are summarized in Fig. 2 and *supp Figure 1*.

**Table 2:** Summary of type-I error and power rate scenarios, with respect to different combinations of sample size, missing rate, and RD rate

Sample size per Arm	Missing rate (%)	RD rate (%)
*N* = 100	10	5, 10, 15
	15	10, 15, 20
	20	15, 20, 25
	25	20, 25, 30
*N* = 200	10	5, 10, 15
	15	10, 15, 20
	20	15, 20, 25
	25	20, 25, 30
*N* = 300	10	5, 10, 15
	15	10, 15, 20
	20	15, 20, 25
	25	20, 25, 30
*N* = 400	10	5, 10, 15
	15	10, 15, 20
	20	15, 20, 25
	25	20, 25, 30

### Power

Datasets for power simulations are generated at the alternative hypothesis that the active treatment is superior to placebo for all post-baseline visits (i.e. ${{\varvec{\beta}}}_{eff}<0$). In addition, trials of longer duration are simulated to mimic the most up-to-date phase 3 antihyperglycemic trials [9, 14, 15] in which a longer duration is needed to reach stable plateau, such that the primary visit of interest is week 40 with week 6, 12, 18, 26 as intermediate post-baseline visits. Using the same notation as above, the model parameters are formulated as below:

$${{\varvec{\beta}}}_{0}=(8, 8, 8, 8, 8, 8)^{\prime}$$

$${{\varvec{\beta}}}_{t}=(0, -0.01, -0.05, -0.1, -0.15, -0.2)^{\prime}$$

$${{\varvec{\beta}}}_{eff}=(0, -0.05, -0.08, -0.12, -0.16, -0.2)^{\prime}$$

RDs in the active group are assumed to experience some regression, i.e., increase in HbA1c, at Week 40 due to off-treatment period. We aim to evaluate the power rate when study dropout rate in both arms is balanced vs unbalanced in subsequent sections.

#### Balanced missing

Rate of missing data (“study dropout”) in each treatment group is the same.

#### Unbalanced missing

Missing data are simulated via a missing not at random (MNAR) mechanism to account for some common study dropout reasons such as efficacy regression after a series of good outcomes [40]. Because participants in the active group are more sensitive to efficacy rebound after sustained improvement, simulated missing rate tends to be higher in the active group and the rates differ by simulation. Further details can be found in the supplementary materials.

Power rate is calculated as the proportion of simulations that are significant at a two-sided 0.05 at Week 40. The average difference of treatment effect is also summarized for each scenario. The 4 MIRD approaches are compared against MMRM and RTB multiple imputation [41, 42].

#### Ratio of RD to dropout rate

Additional power simulations are conducted to understand the impact on power rate when RD rate is a fraction of the dropout rate. Specifically, the following ratios ranging from ¼ to 1 are explored:

- the ratio of RD rate to dropout rate is 1/4
- the ratio of RD rate to dropout rate is 1/3
- the ratio of RD rate to dropout rate is 1/2
- the ratio of RD rate to dropout rate is 1

The sample size and dropout rate scenarios remain the same (according to the first two columns of Table 2). As above, the 4 MIRD approaches are compared against MMRM and RTB multiple imputation. Power rate is calculated as proportion of simulations that are significant at a two-sided alpha of 0.05 at Week 40. The average difference of treatment effect is also summarized for each scenario.

## Data applications

We have applied the MIRD approaches along with MMRM and RTB as reference methods to two phase 3 lipid lowering studies. The primary endpoint is percent change from baseline in LDL-C at Week 52. In studies 1 and 2, a total of 369 and 702 participants are randomized in a 1:1 ratio, respectively. RDs are defined as participants that have discontinued treatment prior to Week 52 but have remained in the study throughout Week 52. Table 3 presents the overall n (%) of missing data and by reasons, and retrieved dropouts by treatment group. Because the majority of missing data are due to adverse event or no longer willing to participate (due to lack of efficacy), the potential for missingness to be related to unobserved data (i.e., MNAR) cannot be ruled out. Therefore, an overall assumption of MNAR is reasonable [43–46]. MIRD analysis results compared to MMRM and RTB are summarized in Tables 4 and 5.

**Table 3:** Disposition of missing data and RDs by treatment group for study 1 and 2

***Study 1***	Placebo (*N* = 184) n (%)	Active (*N* = 185) n (%)
Missing data at Week 52	15 (8.2)	14 (7.6)
* Adverse event*	1	3
* Doesn’t meet entrance criteria*	1	3
* No longer willing to participate*	12	5
* Subject died*	0	1
* Other*	1	2
RDs	19 (10.3)	28 (15.1)
***Study 2***	Placebo (*N* = 348) n (%)	Active (*N* = 354) n (%)
Missing data at Week 52	37 (10.6)	41 (11.6)
* Adverse event*	7	3
* Doesn’t meet entrance criteria*	0	1
* Lost to follow-up*	6	4
* No longer willing to participate*	15	21
* Protocol violation*	0	1
* Subject died*	1	2
* Other*	8	9
RDs	26 (7.5)	47 (13.3)

## Results

### Type-I error simulations

According to Fig. 2, when the dropout rate is low in smaller trials, all MIRD methods tend to be slightly conservative (i.e., with deflated type I error, as illustrated by the red curve) except the newly proposed one-step MCMC approach. The level of deflation tends to lessen with larger sample size. Furthermore, for the smallest sample size scenarios with each arm only randomizing 100 participants and with a dropout rate of 10%, the two two-step approaches are not estimable with 5% RDs. Overall, the three MIRD methods (i.e., except the one-step MCMC approach) have very similar patterns with respect to different scenarios, while the one-step MCMC approach has the most stably controlled type-I error when parameters vary. All four methods have well-controlled type-I error rate in most scenarios, and all are considered as unbiased in all scenarios (*supp Figure 1*).

### Power simulations

**Fig. 3:**
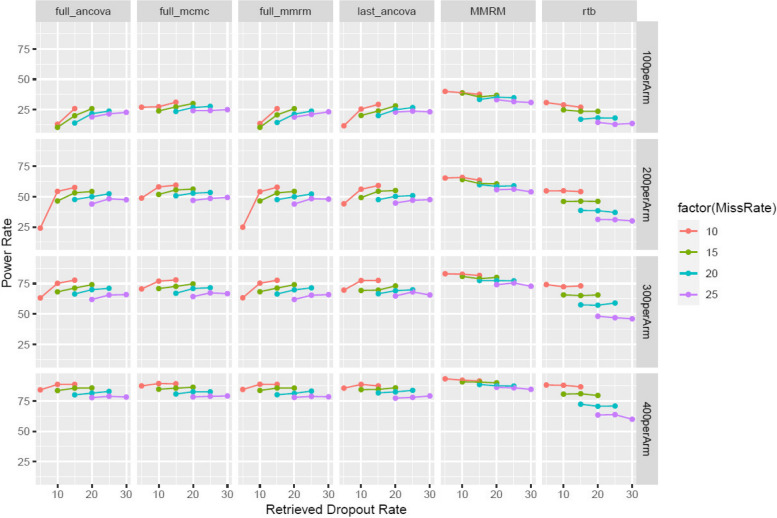
The power rate for a wide variety of scenarios when the study dropout rate is approximately balanced between the two treatment groups (“Balanced Missing”)
  In terms of the estimated difference between the two treatment groups, some extent of attenuation is expected with respect to −0.2 (simulated “ideal” difference of treatment effect with no occurrence of intercurrent events at Week 40) given off-treatment data are used in all methods. According to *supp Figure 2*, all four MIRD approaches as well as the two reference methods lead to attenuated effect sizes at Week 40 following a similar pattern. The attenuation increases when the missing rate goes up. However, among the 6 methods, RTB has the worst attenuation on the treatment effect size as illustrated in a ladder shape in *supp Figure 2*. On the other hand, all four MIRD approaches have very small attenuation, all of which are very comparable to the MMRM.
- *Unbalanced Missing:*
  As described in the simulations, more study dropouts are simulated in the active group than in the placebo group due to the underlying MNAR mechanism. e.g., for scenario with 100 randomized per arm and an overall missing rate of 10%, 1000 simulations will lead to an average missing rate of 14% in the active group versus 6% in the placebo group. Generally, most of the conclusions drawn from “balanced missing” still hold here. The only distinction is for larger trials with more missing data (300, 400 per arm, 20–25% dropout rate), the established MIRD approach based on last on-treatment visit is the most powerful, among all four methods in the MIRD class (Fig. 4).

- *Balanced Missing*: Among the four MIRD approaches, the one-step MCMC approach is the most favorable in all scenarios robustly (Fig. 3). More specifically, the one-step MCMC (class 1(b)) approach is as powerful as the established MIRD approach in most scenarios but is more powerful in scenarios with few RDs. The two two-step approaches (class 1(a)) tend to be less powerful or have non-estimable power rate when the RD rate is low and the trial is relatively small (e.g., 100 per arm, 5% RD rate). Compared to the RTB approach, the class of MIRD methods is more powerful consistently with the power difference more prominent with higher dropout rates. However, all MIRD methods tend to be slightly less powerful than the MMRM analysis which includes off-treatment data.

**Fig. 4:**
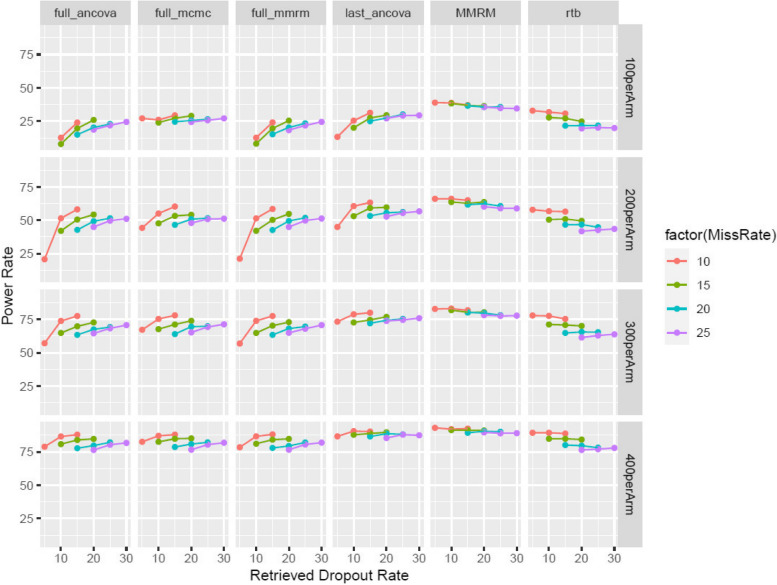
The power rate for a wide variety of scenarios when the study dropout rate is not balanced between the two treatment groups (“Unbalanced Missing”)
- *Ratio of RD to dropout rate:*
  Generally, given a specific scenario (sample size, dropout rate), a higher ratio of RDs to dropouts will lead to improved power rate (according to Fig. 5). Among all four MIRD methods, the one-step MCMC approach is overall more powerful than others. When the ratio is >=½, the one-step MCMC approach is consistently as powerful as or more powerful than the RTB approach. When sample size is increasing, the power difference across all four MIRD approaches diminishes. The one-step MCMC approach and MMRM have the least attenuation of treatment effect across all scenarios (*supp Figure 3*), whereas, other MIRD approaches might have outlying values when sample size is relatively small and RD rate is low (*supp Figure 3*). Generally, RTB approach has more attenuation than other methods and the attenuation gets worse with more missing data. For scenarios of unbalanced missing, the above conclusions still hold according to *supp Figure 5*.

**Fig. 5:**
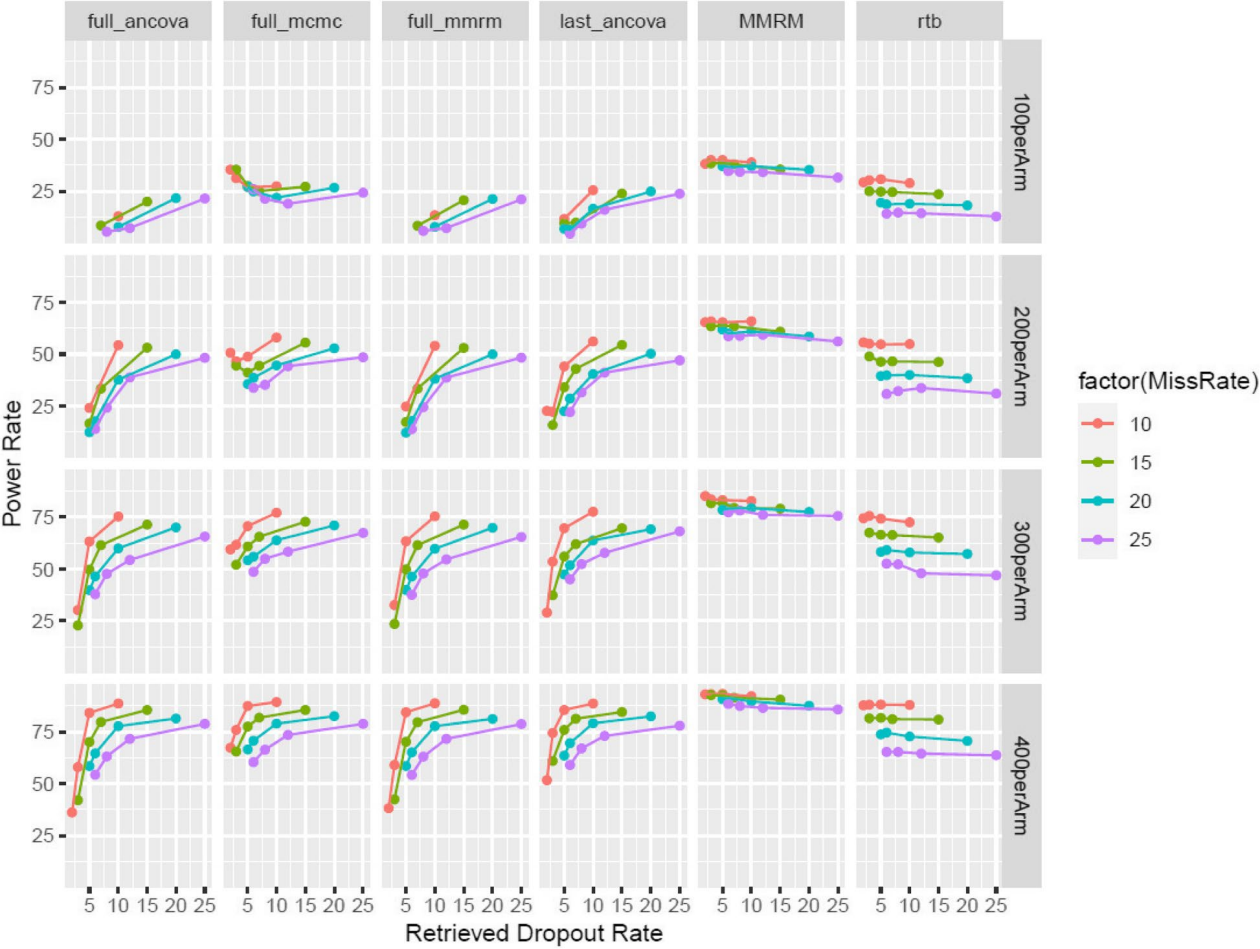
The power rate for a wide variety of scenarios when RD rate is <= the dropout rate, with the ratio ranging from ¼ to 1 (i.e. the points on each curve represent ¼, 1/3, ½ and 1) under “Balanced Missing”

## Data analysis

For study 1, the class of MIRD methods yield very similar placebo-adjusted percent change from baseline in LDL-C at Week 52, with effect size ranging from −46% to −45.6% in point estimate. The effect size of the RTB approach is the smallest while MMRM has the largest effect size, according to Table 4. The observed effect size ranking is consistent with the conclusions from the power simulations with balanced missing rate (Fig. 3*:* red curves of “200 per Arm*).* Because of the drug’s high efficacy, results of all analyses are extremely significant (*p*-value < 0.0001).

**Table 4:** Data analysis results using study 1 dataset

Method	Estimated difference in treatment effect (%)	95% CI for the difference	*p*-value
MIRD methods			
* One-step MCMC (class 1(b))*	−45.6	[−52.2, −39.0]	< 0.0001
* Two-step*^*a*^ *(class 1(a))*	−45.6	[−52.2, −38.9]	< 0.0001
* Established MIRD (class 2)*	−46.0	[−52.4, −39.6]	< 0.0001
Reference methods			
* MMRM*	−48.0	[−53.6, −42.4]	< 0.0001
* RTB*	−45.3	[−51.3, −39.3]	< 0.0001

^a^ANCOVA is used to analyze each of the imputed datasets, due to its equivalence with MMRM on complete dataset. In addition, since there are enough RDs in the dataset, there’s no concern of generating outlying values

Results of analyses using study 2 (Table 5) show a similar ranking: MMRM yields the largest effect size, followed by the MIRD class and RTB. However, the one-step MCMC and two-step approaches, using all available data from RDs return effect sizes almost the same as MMRM but larger than the established MIRD approach. Furthermore, all analyses return very significant results due to high efficacy of the drug.

**Table 5:** Data analysis results using study 2 dataset

Method	Estimated difference in treatment effect (%)	95% CI for the difference	*p*-value
MIRD methods			
* One-step MCMC (class 1(b))*	−46.1	[−52.2, −40.0]	< 0.0001
* Two-step*^*a*^ *(class 1(a))*	−46.1	[−52.1, −40.1]	< 0.0001
* Established MIRD (class 2)*	−43.0	[−48.3, −37.8]	< 0.0001
Reference methods			
* MMRM*	−46.5	[−51.9, −41.1]	< 0.0001
* RTB*	−40.6	[−46.1, −35.1]	< 0.0001

^a^ANCOVA is used to analyze each of the imputed datasets, due to its equivalence with MMRM on complete dataset. In addition, since there are enough RDs in the dataset, there’s no concern of generating outlying values

## Discussion

In this paper we have explored the full class of MIRD analysis for continuous endpoints after it has gained so much popularity over the years in the therapeutic area of metabolic diseases [42]. All methods in this class are based on the same assumption and therefore impute the missing data using RDs. The established MIRD approach (Class 2) only uses last on-treatment visit of RDs to impute the missing data, while the newly proposed approaches (Class 1) use all visits of RDs to impute the missing data. The newly proposed approaches are more straightforward in terms of implementation involving fewer programming derivation steps, and therefore can be more appealing in real applications. The “Retrieved dropouts approach” in Wang’s paper [42] shares similarity with the two-step approach of Class 1 except that in its first step, it creates monotone missingness for the entire dataset whereas in our two-step approach the monotone missingness is solely created out of ${\varvec{\Omega}}$**.** Because 1) the imputation basis of the two-step process should remain the same for consistency and interpretability, and 2) imputed intermediate visits of on-treatment completers from Wang’s approach don’t contribute to either analysis or imputation of missing data, we believe our approach by restricting to ${\varvec{\Omega}}$ (i.e., the union of RDs and missing data) is more reasonable.

The one-step MCMC approach of Class 1 (i.e. class 1(b)) best preserves the type-I error among the two classes combined. It’s also more powerful or as powerful as the established MIRD approach in most scenarios when the RD rate is around the level of dropout rate (Figs. 3 and 4). All four MIRD approaches have similar power rates in settings of large clinical trials. When the missing rate is balanced, the one-step MCMC approach is robustly more powerful than the RTB approach as reflected in Fig. 3, for which this conclusion also holds for the other three MIRD approaches when sample size is increased. For unbalanced settings, this conclusion holds when the RD rate is no lower than the study dropout rate (Fig. 4). In addition, we have further investigated the power rates associated with lower RD rates (with ratio of RD to dropout ranging from ¼ to 1 according to Fig. 5). When the RD rate is at least 1/2 the dropout rate, the one-step MCMC approach is more powerful and therefore should be preferred over RTB, a commonly used sensitivity analysis known for heavily penalizing on premature discontinuations. It has also been shown that when the active group has more study dropouts (*Supp Figure 4*), the power advantage over RTB gets smaller in each scenario. When the RD rate is less than ½ the dropout rate, RTB tends to be more powerful than the class of MIRD approaches.

Because contemporary phase 3 trials in T2D and obesity are usually at least one-year in duration [1–3], trials with longer durations are simulated in this manuscript to reflect the treatment effect over time. In addition, both balanced missing vs unbalanced missing scenarios are simulated to represent trials with or without rescue therapy. For instance, rescue therapy is usually offered in T2D trials and therefore the study dropout rate tends to be very similar between active and placebo groups at the end of the study. Whereas, for trials without rescue therapy, the study dropout rate can vary across the treatment groups. In obesity trials, sometimes the dropout rate in some active group can be higher than the placebo group due to more aggressive titration scheme [47]. When the trial duration is long and rescue therapy is prohibited throughout the trial [2, 11, 12, 17], the dropout rate in placebo group can be higher if participants in the active group are convinced that the benefit of efficacy outweighs tolerability issues. Because the imputation of missing data in the placebo group is generally assumed to follow the MAR assumption, higher dropout rate in placebo group should have very little impact and hence simulations of balanced design should be able to represent this circumstance. An implementation alternative to address this circumstance is to use all available data of the reference group to impute the missing data of the same group, if the underlying MAR assumption is plausible.

Although the basis of imputation is generally defined as the collection of participants who discontinue the treatment prior to the primary visit but remain in the study, the scope of RDs can potentially be broadened for studies with rescue therapy. Specifically, participants who have received rescue therapy but have never discontinued treatment, can potentially be treated as RDs for the following reasons: 1) initiation of rescue therapy is a type of intercurrent event just like treatment discontinuation, so it brings noise in estimating “ideal” treatment effect of randomized arm; 2) participants who have dropped out of the study are likely to try some other diabetes drugs afterwards which resembles the effect of rescue therapy indicating participants with either intercurrent event can be essentially the same in nature. Fundamentally, any decision on the RD scope should be assessed case by case, depending on multiple factors (e.g., study design, sample size, missing data and so on). For instance, if the study can retrieve enough data off treatment and has very low study dropout rate, it might not be worth the efforts to expand the scope.

Our simulation results can be used to guide power prediction of on-going phase 3 trials or to draw conclusions from completed phase 3 study results. In obesity programs that allow rescue therapy [1, 5, 16], the observed disposition is similar to T2D studies which usually have more RDs than missing data (Table 6) implying MIRD analyses tend to be more powerful than other commonly used MNAR sensitivity analyses. But in programs that don’t allow rescue therapy [2, 12, 14], it can be vice versa (Table 7). For instance, Surmont-1 [11] has around 10% missing data in the active groups, but the RD rate is only 1/3 to half of the missing rate across different treatment groups. According to Fig. 5, in the simulated scenario (*N* = 400 per arm, red curve with the ratio ranging between 1/3 and ½) that most mimic this study, the MIRD approach is unlikely to be more powerful than the RTB approach. However, because the drug is overwhelmingly efficacious with *p*-value < 0.001, both approaches will yield the same superiority conclusion. The whole class of MIRD approaches are expected to have bigger treatment effect size (in *Supp Figure 3*) than RTB. In the other example with Surmont-3 [2] which has around 10% missing data in the active group and an RD rate slightly lower than 10%, the simulated scenario (*N* = 300 per arm, the rightmost point on the red curve, Fig. 5) will approximately represent the power of the study: the whole MIRD class is more powerful than RTB with larger effect size (*Supp Figure 3*).

**Table 6:** Disposition summary of some completed phase 3 T2D studies

Study Name	Compound dose	Number of RDs^a^	Number of study dropouts	Number per Arm in mITT
Surpass-2 (T2D)	Tirzapetide 5 mg	25	14	470
	Tirzapetide 10 mg	42	16	469
	Tirzapetide 15 mg	45	17	470
	Sema 1 mg	23	18	469
Surpass-3 (T2D)	Tirzapetide 5 mg	22	20	358
	Tirzapetide 10 mg	37	29	360
	Tirzapetide 15 mg	44	15	359
	insulin degludec	9	31	360

^a^Calculated as difference between # participants who discontinue treatment prior to the primary visit- # participants who discontinue the study prior to the primary visit

**Table 7:** Disposition summary of some completed phase 3 CWM/obesity studies

Study Name	Compound dose	Number of RDs^a^	Number of study dropouts	Number per Arm in mITT
Surmount-1	Tirzapetide 5 mg	21	69	630
	Tirzapetide 10 mg	30	74	636
	Tirzapetide 15 mg	31	64	630
	placebo	22	148	643
Surmount-2 (allows diabetes rescue)	Tirzapetide 10 mg	13	16	312
	Tirzapetide 15 mg	14	29	311
	Placebo	13	34	315
Surmount-3	Tirzapetide MTD	26	35	287
	Placebo	24	65	292

^a^Calculated as difference between # participants who discontinue treatment prior to the primary visit- # participants who discontinue the study prior to the primary visit

Missing data due to adverse events and no longer willing to participate (due to lack of efficacy) constituting more than half of the missing data of each dataset, justifies the use of methods based on appropriate MNAR assumptions in the section of data analysis. The two datasets from the data analysis section well demonstrate the performance of the MIRD methods compared to the reference methods from different angles. Consistent with the conclusions drawn from power simulations, it’s noteworthy that the power gain from larger sample size seems to overweigh the power loss when RD rates are slightly lower than missing rate. Specifically, study 2 has larger sample size than study 1 (approximately 350 vs 200 per arm). However, study 1 has slightly higher RD rate in both arms and more importantly, the RD rate in both arms of study 1 is higher than the missing rate while the RD rate in the placebo group of study 2 is slightly lower than the missing rate. Results of the newly proposed MIRD methods applied to study 2 datasets are quite on par with the on-study MMRM results according to Table 5 which manifest the conclusion that larger sample size will further improve power on top of decent RD rate (around the same level or higher than missing rate). MMRM is technically applicable to longitudinal data with observations that occur after intercurrent events included in the analysis (i.e., under treatment policy strategy) and can be more powerful in certain circumstances. However, the underlying caveat is it’s considered as a statistical approach targeting the on-treatment hypothetical estimand due to its inherent MAR assumption. Therefore, it is not considered compatible with the treatment policy estimand.

## Conclusion

The newly proposed MIRD approaches along with the established MIRD approach constitute a full landscape of retrieved dropout-based approaches for continuous endpoints in clinical trials. The newly proposed approaches, based on all visits of RDs, are easier to implement. However, the established MIRD approachcan be more powerful than the two-step approaches in certain scenarios. Overall, the one-step MCMC approach is the most recommended if the ratio of RD to missing data is greater than ½. It’s important to note any of the retrieved dropout analyses shouldn’t be forced on trials that don’t have sufficient RDs or not designed to collect RDs, in which circumstances approaches based on other assumptions should be considered.

## Supplementary Information

Supplementary Material 1.

## Data Availability

The dataset used in the data analysis won’t be available from corresponding author, due to Pfizer’s policy and obligation to protect patients’ privacy. The datasets used in the simulation studies can be made available from corresponding author on reasonable request.
