# Supplementary material for: Comprehensive implementations of multiple imputation using retrieved dropouts for continuous endpoints
Source: BMC Med Res Methodol. 2025 Feb 21;25:47. doi: 10.1186/s12874-025-02494-5 (PMC11846319; doi:10.1186/s12874-025-02494-5)
Supplement: Supplementary file 1 — Supplementary Material 1. [file 12874_2025_2494_MOESM1_ESM.docx]

Contents

[Example SAS code 1](#_Toc165288707)

[Simulations 2](#_Toc165288708)

[Covariance structure 2](#_Toc165288709)

[Missing not at Random 2](#_Toc165288710)

[Figures 3](#_Toc165288711)

#

# Example SAS code

The adeff dataset (ADaM type) is in long format for which we use the following sample dataset to illustrate:

| subjid | trt01pn | base | avisit | avisitn | aval | &rd |
| --- | --- | --- | --- | --- | --- | --- |
| 10011001 | 1 | 8.5 | baseline | 0 | 8.5 | 0 |
| 10011001 | 1 | 8.5 | week 4 | 4 | 8 | 0 |
| 10011001 | 1 | 8.5 | week 8 | 8 | 8.2 | 0 |
| 10011001 | 1 | 8.5 | week 12 | 12 | 8.3 | 0 |
| 10011001 | 1 | 8.5 | week 18 | 18 | 7.6 | 0 |
| 10011001 | 1 | 8.5 | week 26 | 26 | 7 | 0 |
| 10011002 | 1 | 8.6 | baseline | 0 | 8.6 | 0 |
| 10011002 | 1 | 8.6 | week 4 | 4 | 8.05 | 0 |
| 10011002 | 1 | 8.6 | week 8 | 8 | 8.15 | 0 |
| 10011002 | 1 | 8.6 | week 12 | 12 | 8.28 | 0 |

- Class 1(a): Two-step approach:

*Because the ADaM dataset comes in long format, first it needs to be transposed to wide format;

proc transpose data=adeff prefix=v out=adeff_t_new;

by subjid trt01pn base &rd/*retrieve dropout status*/ &add_var /*additional covariates needed for ANCOVA*/;

id &avisitn;

var aval;

run;

*STEP1: Monotone missingness is created our of Omega (RD or subjects with missing primary visit) by treatment group;

proc mi data=adeff_t_new(where=(&rd=1 or &primary_time=.)) seed=&seed_monotone nimpute=&nimpute out=adeff_mi_step1;

mcmc impute=monotone;

by trt01pn;

var base &intermediate_time &primary_time;

run;

*STEP2: Regression-based multiple imputation;

proc sort data=adeff_mi_step1;

by _imputation_ trt01pn;

run;

proc mi data=adeff_mi_step1 out=adeff_mi2 nimpute=1 seed=&seed;

*class trt01pn ;

by _imputation_ trt01pn ;

monotone reg;

var base &intermediate_time &primary_time;

run;

- Class 1(b): One-step MCMC approach:

proc mi data=adeff_t_new(where=(&rd=1 or &primary_time=.)) seed=&seed nimpute=&nimpute out=adeff_mi2_mcmc;

mcmc chain=multiple displayinit initial=em(maxiter=1000);

by trt01pn;

var base &intermediate_time &primary_time;

run;

- Class 2: Established MIRD approach:

*v_last denote value of the last on-treatment visit;

proc mi data=adeff_t_new(where=(&rd=1 or &primary_time=.)) out=adeff_mi nimpute=&nimpute seed=&seed;

*class trt01pn ;

by trt01pn ;

monotone reg;

var base v_last &primary_time;

run;

# Simulations

## Covariance structure

Model specification of Covariance structure in the simulations:

- Type-I error/Power: The diagonal elements (variance of HbA1c at any time point) are 1 and the off-diagonal elements (covariance of any two time points) are set to be 0.6.

## Missing not at Random

The mechanism to simulate missing not at random in power simulations:

- subjects who initially see improvements (defined as <baseline) up to the middle point of the trial (defined as Week 12 or later) and then start to get worse (defined as >=baseline) afterwards are more likely to drop out of the study.

e.g., the following subject initially sees improvement in A1c (%) at week 6 and 12 then his week 18 value is worse than baseline, so he/she is part of the pool of subjects that are more likely to drop out after Week 18.

| Baseline | Week 6 | Week 12 | Week 18 | Week 26 | Week 40 |
| --- | --- | --- | --- | --- | --- |
| 6.88757 | 6.79616 | 6.73212 | 6.976961 | 7.338479 | 6.22189 |

- Subjects who initially observe “more” improvements (defined as <0.98*baseline) in A1c compared to their baseline up to later stage (defined as Week 18 or later) of the trial, and then lose the trend afterwards (defined as >=0.98*baseline) are more likely to drop out of the study.

e.g., the following subject initially sees “more” improvement defined as (A1c <0.98 * baseline) consistently till Week 18. However, he/she loses the trend at Week 26. Hence, he/she is more likely to drop out after Week 26.

| Baseline | Week 6 | Week 12 | Week 18 | Week 26 | Week 40 |
| --- | --- | --- | --- | --- | --- |
| 7.680987 | 7.492599 | 6.671825 | 7.070184 | 7.545417 | 7.515658 |

According to the pool defined above, <% missing rate* sample size > are randomly selected from this pool and the last on-treatment visit is determined by the algorithm above.

# Figures


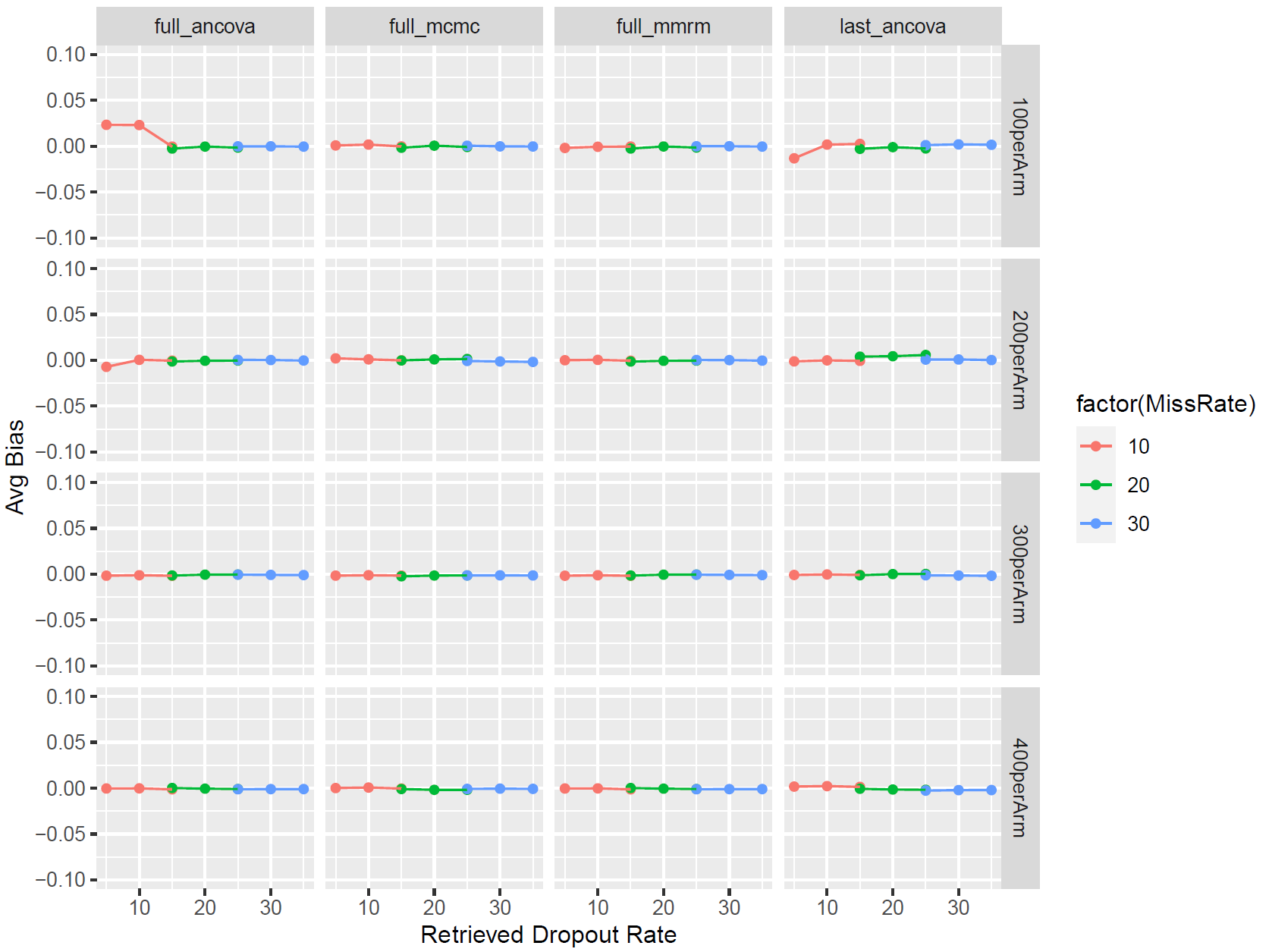


***SuppFigure 1****. Average bias of difference of treatment effect (benchmark is 0) at Week 26 from type-I error simulations*


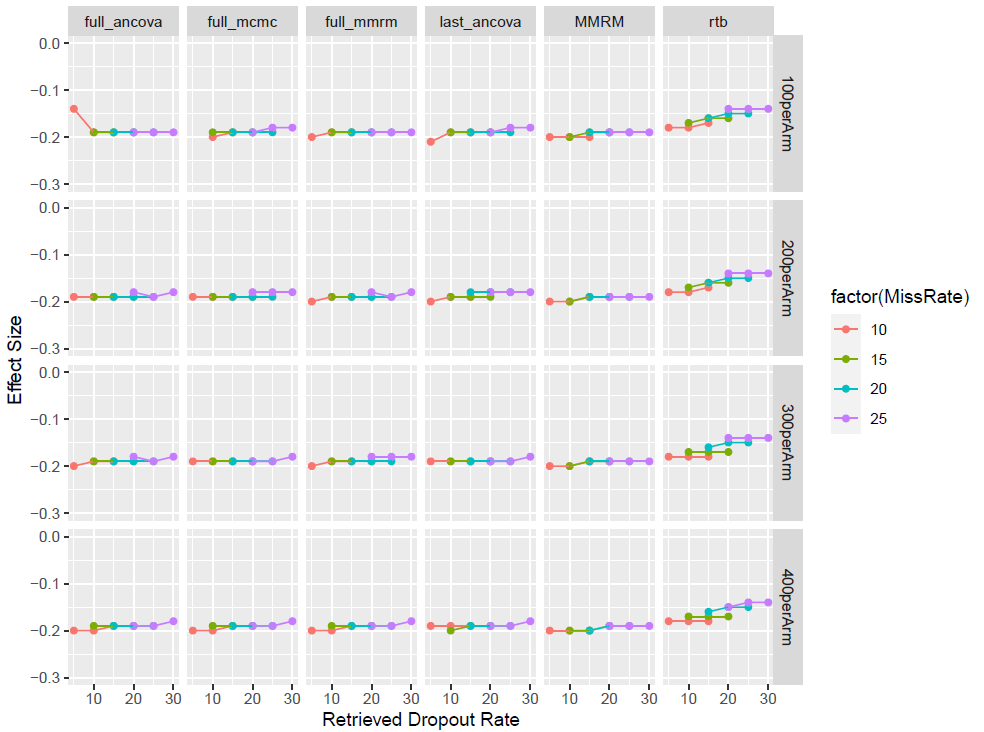


***SuppFigure 2****. Average effect size of difference of treatment effect (benchmark is -0.2) at Week 40 from power simulations, assuming the missing rate is balanced across the two treatment groups*


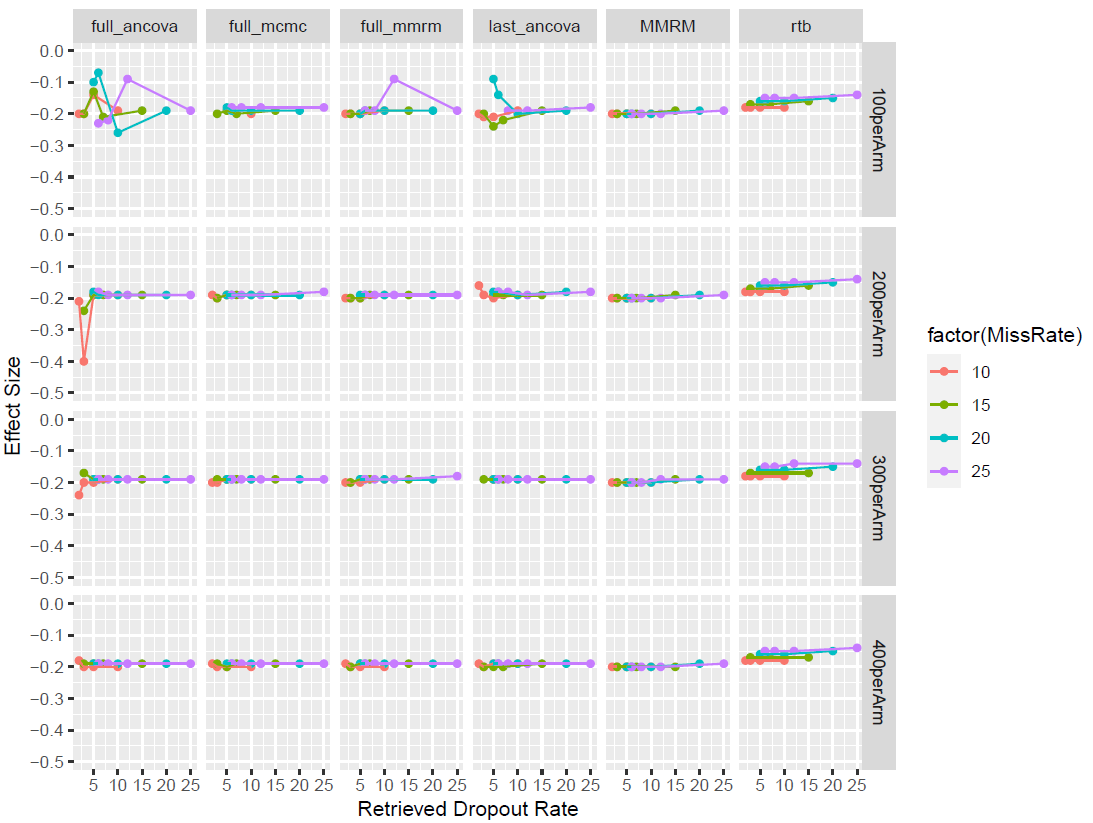


***SuppFigure 3****. Average effect size of difference of treatment effect (benchmark is -0.2) at Week 40 from power simulations, when RD rate is <= the dropout rate, with the ratio ranging from ¼ to 1 (i.e. the points on each curve represent ¼, 1/3, ½ and 1) under “Balanced Missing”.*


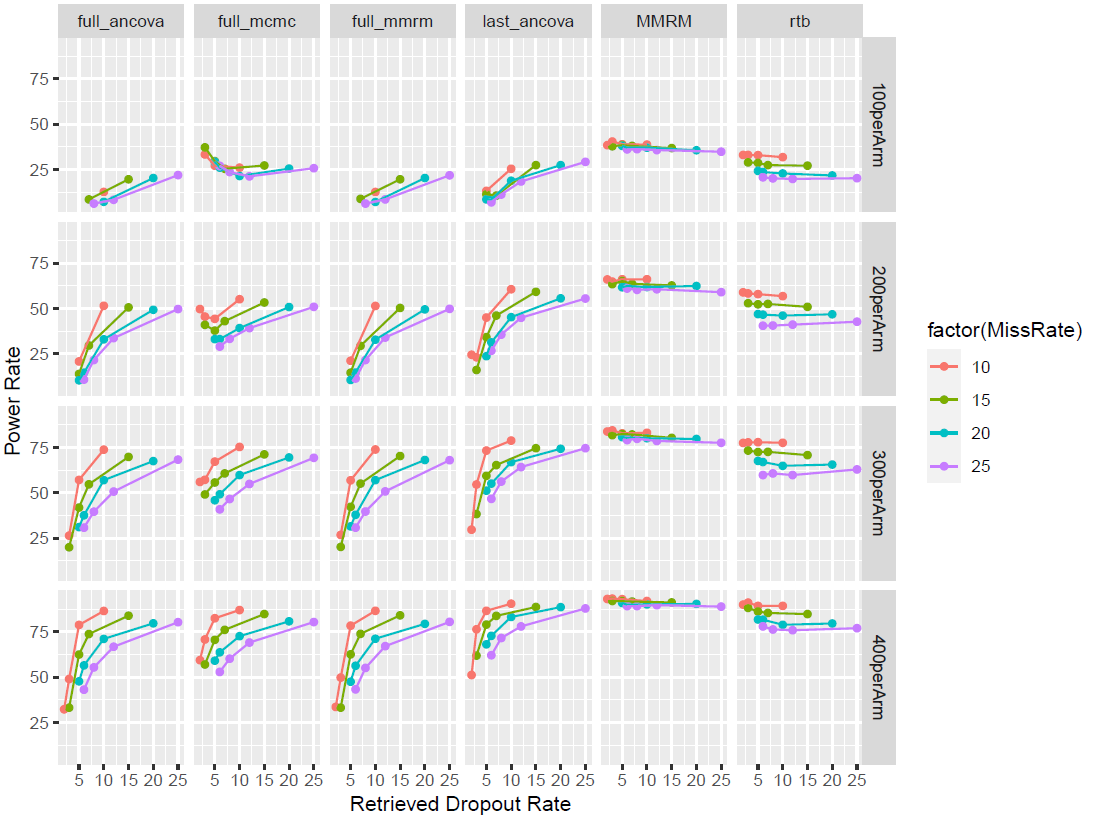


***SuppFigure 4****. the power rate for a wide variety of scenarios when RD rate is <= the dropout rate, with the ratio ranging from ¼ to 1 (i.e. the points on each curve represent ¼, 1/3, ½ and 1) under “Unbalanced Missing”.*


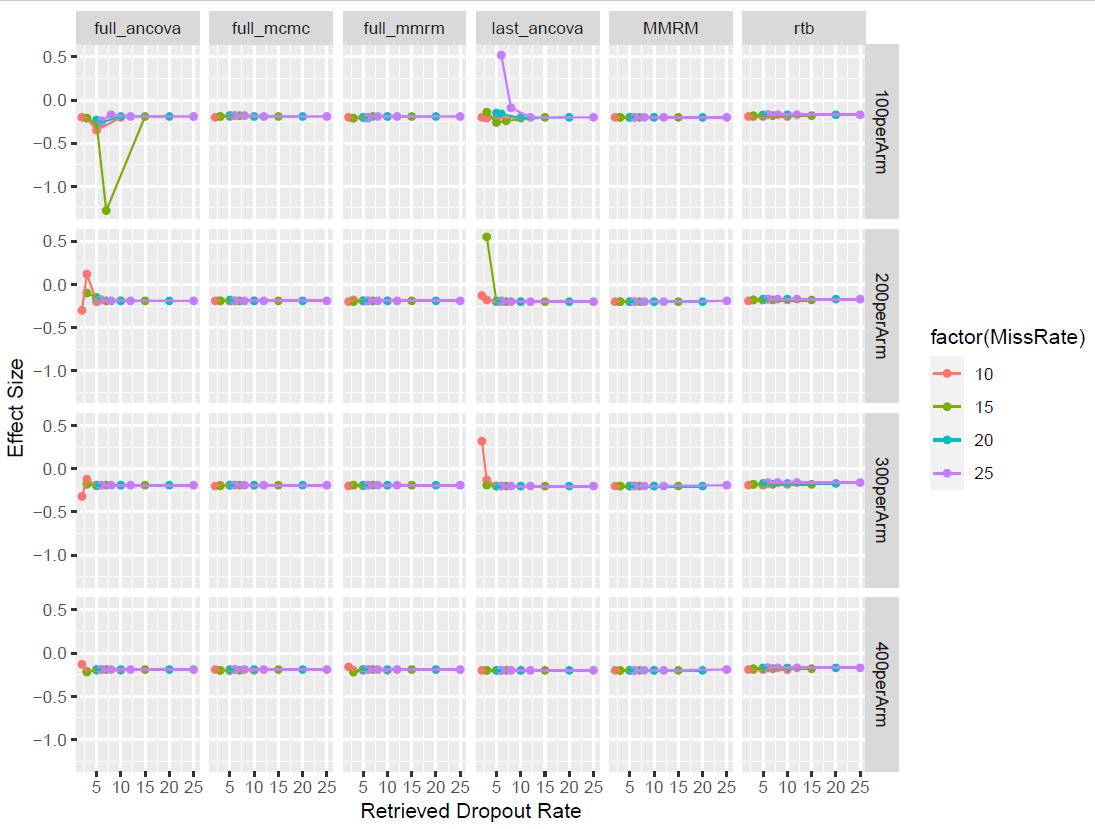


***SuppFigure 5****. Average effect size of difference of treatment effect (benchmark is -0.2) at Week 40 from power simulations, when RD rate is <= the dropout rate, with the ratio ranging from ¼ to 1 (i.e. the points on each curve represent ¼, 1/3, ½ and 1) under “Unbalanced Missing”.*


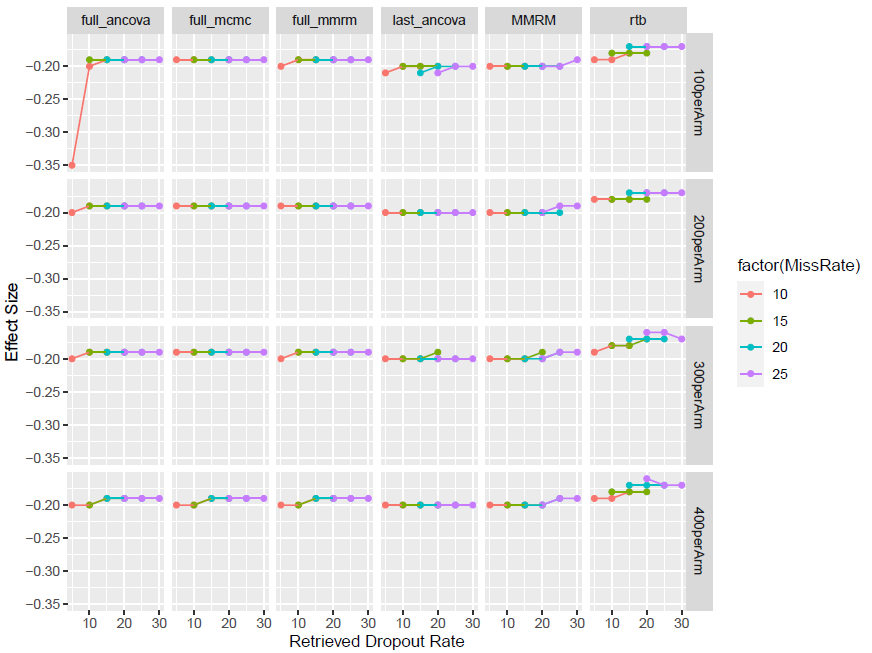


***SuppFigure 6****. Average effect size of difference of treatment effect (benchmark is -0.2) at Week 40 from power simulations, assuming the missing rate is unbalanced across the two treatment groups*
